# Population-Based Assessment of HPV Genotype-Specific Cervical Cancer Survival: CDC Cancer Registry Sentinel Surveillance System

**DOI:** 10.1093/jncics/pky036

**Published:** 2018-08-11

**Authors:** Benjamin D Hallowell, Mona Saraiya, Trevor D Thompson, Elizabeth R Unger, Charles F Lynch, Tom Tucker, Glenn Copeland, Brenda Y Hernandez, Edward S Peters, Edward Wilkinson, Marc T Goodman, for the HPV Typing of Cancers Workgroup

**Affiliations:** 1 Centers for Disease Control and Prevention, Atlanta, GA; 2 University of Iowa, Iowa City, IA; 3 University of Kentucky College of Public Health, Lexington, KY; 4 Michigan Department of Community Health, Lansing, MI; 5 University of Hawaii, Honolulu, HI; 6 Health Science Center School of Public Health, Lousiania State University, New Orleans, LA; 7 Department of Pathology, University of Florida College of Medicine, Gainesville, FL; 8 Cedars Sinai Medical Center, Los Angeles, CA

## Abstract

**Background:**

Human papillomavirus (HPV) genotype influences the development of invasive cervical cancer (ICC); however, there is uncertainty regarding the association of HPV genotype with survival among ICC patients.

**Methods:**

Follow-up data were collected from 693 previously selected and HPV-typed ICC cases that were part of the Centers for Disease Control and Prevention Cancer Registry Surveillance System. Cases were diagnosed between 1994 and 2005. The Kaplan-Meier method was used to estimate five-year all-cause survival. A multivariable Cox proportional hazards model was used to estimate the effect of HPV genotype on survival after adjusting for demographic, tumor, and treatment characteristics.

**Results:**

Five-year all-cause survival rates varied by HPV status (HPV 16: 66.9%, HPV 18: 65.7%, HPV 31/33/45/52/58: 70.8%, other oncogenic HPV genotypes: 79.0%, nononcogenic HPV: 69.3%, HPV-negative: 54.0%). Following multivariable adjustment, no statistically significant survival differences were found for ICC patients with HPV 16–positive tumors compared with women with tumors positive for HPV 18, other oncogenic HPV types, or HPV-negative tumors. Women with detectable HPV 31/33/33/45/52/58 had a statistically significant 40% reduced hazard of death at five years (95% confidence interval [CI] = 0.38 to 0.95), and women who tested positive for nononcogenic HPV genotypes had a statistically significant 57% reduced hazard of death at five years (95% CI = 0.19 to 0.96) compared with women with HPV 16 tumors. Few statistically significant differences in HPV positivity, tumor characteristics, treatment, or survival were found by race/ethnicity.

**Conclusions:**

HPV genotype statistically significantly influenced five-year survival rates among women with ICC; however, screening and HPV vaccination remain the most important factors to improve patient prognosis and prevent future cases.

Despite declines in the burden of cervical cancer since the 1930s, as a result of screening and improved treatment, 12_ _578 new cases and 4115 deaths occurred in 2014 in the United States (1). Human papillomavirus (HPV) is the primary cause of invasive cervical cancer (ICC) (2,3). Although cervical infection with HPV is common, it is thought that about 70% of new HPV infections resolve within one year and 90% resolve within two years without treatment (4). Persistent HPV infection with high-risk (oncogenic) HPV types can result in cancer, however, and our previous work has found that 91% of cervical cancers in the United States were positive for HPV DNA and could be attributed to HPV (5,6). Although multiple oncogenic HPV types can cause cancer, about 70% of cervical cancers are attributable to HPV 16 and HPV 18 (3,5–9).

Several demographic, clinical, and HPV-related factors have been associated with survival among cervical cancer patients (10–17). Demographic factors associated with poorer survival rates include African American (10,11,17,18) and Hispanic race/ethnicity (12) and residence in rural areas (10). Clinically, lymph node involvement (13), larger tumor size (13), more advanced tumor stage (14,15,17), older age at diagnosis (13–15,17), and receipt of radiation/chemotherapy when compared with surgery (13) predict poorer survival outcomes in ICC patients. African American women are more likely to present with advanced-stage tumors at diagnosis (10,11,16, 18) and are less likely to receive treatment (11,16) when compared with white women. These clinical differences appear to explain some, but not all, of the impact of race on ICC survival (11,15,16).

A previous study examining the effect of HPV genotype on ICC survival found that women with HPV 16/18–positive tumors had worse survival than women whose tumors were positive for other HPV types (19). However, smaller institution-based studies have failed to find statistically significant survival differences between ICC patients by HPV genotype in multivariable models (13,20–23). Further complicating these analyses is the observation that HPV-negative cervical tumors are more frequent among older women, diagnosed at more advanced stages, more common among non-Hispanic white women, and more likely to be adenocarcinoma or endometrioid histology when compared with HPV-positive tumors (5,19,24). Although some HPV-negative results may be due to technical limitations in testing or to misclassification of cancers arising in lower–uterine segment endometrium as endocervical origin, these tumors may also represent distinct entities.

The objective of the present study is to overcome some of the limitations of previous investigations to better characterize the impact of HPV genotype on ICC survival with follow-up data from a large sample of ICC patients from population-based cancer registries in the United States (5,6).

## Methods

A simple random sample of invasive cervical cancer patients diagnosed from 1994 to 2005 was performed as part of the Centers for Disease Control Cancer Registry Sentinel Surveillance System (5). Participants were selected from seven central, population-based cancer registries: Florida, Kentucky, Louisiana, Michigan, Hawaii, Los Angeles, and Iowa. Participating registries collect information from hospitals, pathology laboratories, and treatment facilities to obtain information on cancer diagnosis, tumor characteristics, and cancer treatment. Cancer registry data are routinely linked with other databases (eg, National Death Index, Equifax) to obtain accurate vital status follow-up for all individuals diagnosed with cancer in their defined catchment area.

Protocols for identifying and submitting the formalin-fixed paraffin-embedded tissue samples were identical across all participating registries and have been described previously (5). Study eligibility included cases diagnosed between 1994 and 2005 with histologically confirmed ICC (ICD-O-3 site codes C53.0, C53.1, C53.8, C53.9, and behavior code 3).

Of the 786 invasive cervical cancer tissue samples that were eligible for testing, tumor tissues from 777 patients were adequate for evaluation and were typed for HPV. Specimens from the Los Angeles Cancer Registry (70 ICC cases) were excluded because of missing follow-up data. Fourteen additional tissue samples with histologies other than squamous cell carcinoma and adenocarcinoma were also excluded (small cell/neuroendocrine, n = 3, other specified carcinomas, n = 4, and noncarcinomas, n = 7) resulting in a final sample size of 693 unique tumors.

The institutional review board for the Centers for Disease Control and Prevention and each participating registry approved this study.

### DNA Extraction and HPV Typing

All laboratory methods have been previously described (5). Initial HPV genotyping was performed on all samples using the Linear Array HPV Genotyping Test, followed by INNO-LiPA HPV Genotyping Assay (Innogenetics) for negative or inadequate results.

### Statistical Methods

Patient and tumor characteristics were compared by race and according to six hierarchical HPV status groups, with HPV 16 as the most oncogenic type: 1) HPV 16–positive; 2) HPV 16–negative, HPV 18–positive; 3) HPV 16/18–negative, HPV 31/33/45/52/58–positive; 4) HPV 16/18/31/33/45/52/58–negative, positive for other oncogenic HPV types not covered by the nonavalent vaccine (35/39/51/56/59/66/68); 5) negative for all oncogenic HPV types, positive for nononcogenic HPV types (6/11/26/40/42/43/44/53/54/55/61/62/64/67/69/70/71/72/73/74/81/82/83/84/89/IS39/X); and 6) negative for all HPV types. Individuals with multiple HPV infections were assigned the first group in which they were eligible going down the hierarchical categories (eg, an HPV 16– and 18–positive tumor would be placed in group 1). Continuous variables are presented as medians and 25th/75th percentiles, and discrete variables as frequencies and percentages. Statistical testing was performed using the likelihood ratio chi-square test for discrete variables. The Kruskal-Wallis test was used to test for differences among continuous variables. Five-year survival curves are presented as Kaplan-Meier estimates. Statistical testing for differences in unadjusted survival rates across patient and tumor characteristics was performed using a two-sided log-rank test.

A time-dependent Cox proportional hazards model was used to determine the independent predictors of five-year survival. Age, race/ethnicity, stage, grade, histology type, HPV status, surgery, radiation, and chemotherapy were included as covariates in the survival model. Time-dependent covariates for the treatment variables were used to mitigate artificial inflation of the effect estimates associated with survival. For each of these treatments, patients were considered untreated until the date of treatment. The linearity assumption for the continuous age variable was assessed using restricted cubic spline functions. Missing data were imputed for all independent predictors except treatment, using the aregImpute function in R. The aregImpute function performs multiple imputation using predictive mean matching. Due to the time-dependent nature of the treatment variables, observations missing treatment status or timing were excluded from the multivariable analysis.

To determine if our sample was representative of the general US population, we compared HPV-typed ICCs with nontyped ICCs in the general population by age and race. ICCs were representative of nontyped ICCs by age and were not representative of nontyped patients by race due to an overselection of nonwhite ICCs to assist with the analysis of HPV prevalence by increasing the sample size in these populations (6).

## Results

In this population-based study of 693 ICC cases, we found that 91.5% of tumors tested positive for HPV (Table 1). Based on our hierarchical classification, 51.4% of tumors tested positive for HPV 16, 15.9% for HPV 18, 14.6% for HPV 31/33/45/52/58 (additional oncogenic HPV types covered in the nonavalent vaccine), 6.3% for other oncogenic HPV types, 3.3% for nononcogenic HPV types, and 8.5% were HPV-negative. The average age at ICC diagnosis varied by HPV status (*P* < .0001), particularly when comparing ICCs that tested positive for HPV 16/18 (median age = 45 years) with tumors positive for other HPV types (median age = 47–54 years) and those with no HPV infection detected (median age = 57 years). Adenocarcinomas were statistically significantly more likely among women whose tumors tested positive for HPV 18 (47.3%) and among women with HPV-negative tumors (56.1%) when compared with women whose tumors tested positive for other HPV types (*P* < .0001). No statistically significant differences were observed among races or by tumor stage, grade, or treatment types when stratified by the six hierarchical HPV groupings. There were higher proportions of women with HPV-negative tumors had a higher proportion of tumors that were treated with chemotherapy (53.8%) and were poorly/undifferentiated (59.2%) and at distant stage (17%) at diagnosis when compared with women with HPV-positive tumors, but these differences were not statistically significant.

**Table 1.** Distribution of patient and tumor characteristics by hierarchical HPV positivity status among invasive cervical cancer patients (n = 693)

Characteristic	HPV 16 (n = 356)	HPV 18 (n = 110)	HPV 31/33/45/52/58 (n = 101)	Other oncogenic HPV types (n = 44)	Nononcogenic HPV types (n = 23)*	HPV-negative (n = 59)	*P*†
Age, y‡	37/45/56	38/45/53	37/50/61	42/47/62	49/54/69	46/57/72	<.0001
	48.1 ± 15.2	46.9 ± 13.1	50.8 ± 16.4	51.4 ± 15.5	59.3 ± 16.2	58.6 ± 16.9	
Race, No. (%)							.0773
White non-Hispanic	199 (56.5)	62 (56.9)	44 (43.6)	24 (54.5)	12 (52.2)	39 (66.1)	
Black non-Hispanic	62 (17.6)	22 (20.2)	18 (17.8)	12 (27.3)	3 (13.0)	4 (6.8)	
Hispanic	43 (12.2)	9 (8.3)	17 (16.8)	6 (13.6)	3 (13.0)	7 (11.9)	
Other	48 (13.6)	16 (14.7)	22 (21.8)	2 (4.5)	5 (21.7)	9 (15.3)	
Stage, No. (%)							.0689
Localized	179 (53.8)	62 (63.3)	51 (54.3)	23 (56.1)	9 (40.9)	19 (35.8)	
Regional	120 (36.0)	25 (25.5)	38 (40.4)	15 (36.6)	10 (45.5)	25 (47.2)	
Distant	34 (10.2)	11 (11.2)	5 (5.3)	3 (7.3)	3 (13.6)	9 (17.0)	
Grade (differentiation), No. (%)							.0696
Well	32 (12.6)	13 (15.3)	2 (3.0)	2 (5.7)	2 (11.8)	4 (8.2)	
Moderately	110 (43.3)	30 (35.3)	32 (47.8)	21 (60.0)	7 (41.2)	16 (32.7)	
Poorly/undifferentiated	112 (44.1)	42 (49.4)	33 (49.3)	12 (34.3)	8 (47.1)	29 (59.2)	
Histology, No. (%)							<.0001
Adenocarcinoma	56 (16.1)	52 (47.3)	12 (12.1)	3 (6.8)	1 (4.5)	32 (56.1)	
Squamous cell carcinoma	292 (83.9)	58 (52.7)	87 (87.9)	41 (93.2)	21 (95.5)	25 (43.9)	
Surgery, No. (%)	216 (62.6)	77 (74.0)	62 (61.4)	28 (65.1)	12 (52.2)	33 (61.1)	.2260
Radiation, No. (%)	159 (46.1)	44 (41.9)	40 (40.8)	20 (48.8)	14 (60.9)	32 (58.2)	.2061
Chemotherapy, No. (%)	105 (32.2)	29 (29.9)	30 (30.0)	15 (38.5)	8 (36.4)	28 (53.8)	.0533

* Nononcogenic HPV types included: (6/11/26/40/42/43/44/53/54/55/61/62/64/67/69/70/71/72/73/74/81/82/83/84/89/IS39/X). Frequencies may not sum to column totals due to missing data for the row characteristics. HPV = human papillomavirus.

† Statistical testing performed using Kruskal-Wallis rank-sum test for continuous variables and likelihood ratio chi-square test for discrete variables.

‡ Continuous variables presented as lower quartile/median/upper quartile and mean ± standard deviation.

Few statistically significant differences in HPV positivity, tumor characteristics, or treatment were found by race/ethnicity (Table 2). Non-Hispanic black women had the highest proportion of squamous cell carcinomas (90.0% vs 71.8%–78.8%, *P* = .0002), although their proportion of HPV 18–positive ICC was similar to that of other women. They also had the highest proportion of distant-stage tumors (15.0% vs 7.0%–10.3%), the lowest proportion of women receiving surgery (54.7% vs 59.8%–67.8%; a treatment associated with earlier detection/treatment), and the highest proportion of women receiving chemotherapy (42.1% vs 26.7%–40.6%), but these differences were not statistically significant.

**Table 2.** Distribution of patient and tumor characteristics by race/ethnicity among invasive cervical cancer patients (n = 688)*

Characteristic	White non-Hispanic (n = 380)	Black Non-Hispanic (n = 121)	Hispanic (n = 85)	Other (n = 102)	*P*†
Age, y†	38/47/60	38/49/59	38/45/56	37/47/62	.9039
	49.8 ± 15.9	50.1 ± 15.8	48.4 ± 13.0	50.8 ± 16.9	
Stage, No. (%)					.2458
Localized	195 (55.6)	48 (44.9)	39 (49.4)	57 (57.0)	
Regional	120 (34.2)	43 (40.2)	34 (43.0)	36 (36.0)	
Distant	36 (10.3)	16 (15.0)	6 (7.6)	7 (7.0)	
Grade, No. (%)					.2622
Well differentiated	28 (10.0)	7 (7.7)	11 (18.0)	9 (12.7)	
Moderately differentiated	117 (41.6)	40 (44.0)	30 (49.2)	28 (39.4)	
Poorly differentiated/undifferentiated	136 (48.4)	44 (48.4)	20 (32.8)	34 (47.9)	
Histology, No. (%)					.0002
Adenocarcinoma	105 (28.2)	12 (10.0)	18 (21.2)	21 (21.4)	
Squamous cell carcinoma	267 (71.8)	108 (90.0)	67 (78.8)	77 (78.6)	
Surgery, No. (%)	246 (67.8)	64 (54.7)	53 (63.9)	61 (59.8)	.0633
Radiation, No. (%)	172 (48.0)	60 (49.6)	38 (46.3)	38 (37.6)	.2562
Chemotherapy, No. (%)	111 (32.0)	48 (42.1)	28 (40.6)	27 (26.7)	.0543

* Five individuals with missing race/ethnicity were excluded. Frequencies may not sum to column totals due to missing data for the row characteristics.

† Continuous variables presented as lower quartile/median/upper quartile and mean ± standard deviation.

‡ Statistical testing performed using Kruskal-Wallis rank-sum test for continuous variables and likelihood ratio chi-square test for discrete variables.

Adenocarcinomas were more likely to be well differentiated at diagnosis when compared with squamous cell carcinomas (*P* = .0008) (Table 3). Women diagnosed with squamous cell carcinoma were statistically significantly more likely to be treated using radiation (49.4% vs 37.3%) and were statistically significantly less likely to be treated using surgery (58.9% vs 80.1%) when compared with women diagnosed with adenocarcinoma. No statistically significant differences by histology were observed for chemotherapy treatment (Table 3).

**Table 3.** Distribution of patient and tumor characteristics by histology among invasive cervical cancer patients (n = 680)*

Characteristic	Squamous cell carcinoma (n = 524)	Adenocarcinoma (n = 156)	*P*†
Age, y†	38/47/59	36/47/60	.2924
	50.0 ± 15.5	48.7 ± 16.0	
Race, No. (%)			.0002
White non-Hispanic	267 (51.4)	105 (67.3)	
Black non-Hispanic	108 (20.8)	12 (7.7)	
Hispanic	67 (12.9)	18 (11.5)	
Other	77 (14.8)	21 (13.5)	
Stage, No. (%)			.2099
Localized	250 (51.4)	86 (59.7)	
Regional	186 (38.3)	45 (31.3)	
Distant	50 (10.3)	13 (9.0)	
Grade, No. (%)			.0008
Well differentiated	29 (7.8)	26 (20.3)	
Moderately differentiated	170 (45.5)	46 (35.9)	
Poorly differentiated/undifferentiated	175 (46.8)	56 (43.8)	
Surgery, No. (%)	298 (58.9)	121 (80.1)	<.0001
Radiation, No. (%)	249 (49.4)	56 (37.3)	.0089
Chemotherapy, No. (%)	172 (35.8)	40 (27.6)	.0627

* Thirteen individuals with missing histology were excluded. Frequencies may not sum to column totals due to missing data for the row characteristics.

† Continuous variables presented as lower quartile/median/upper quartile and mean ± standard deviation.

‡ Statistical testing performed using Kruskal-Wallis rank-sum test for continuous variables and likelihood ratio chi-square test for discrete variables.

**Figure 1.**
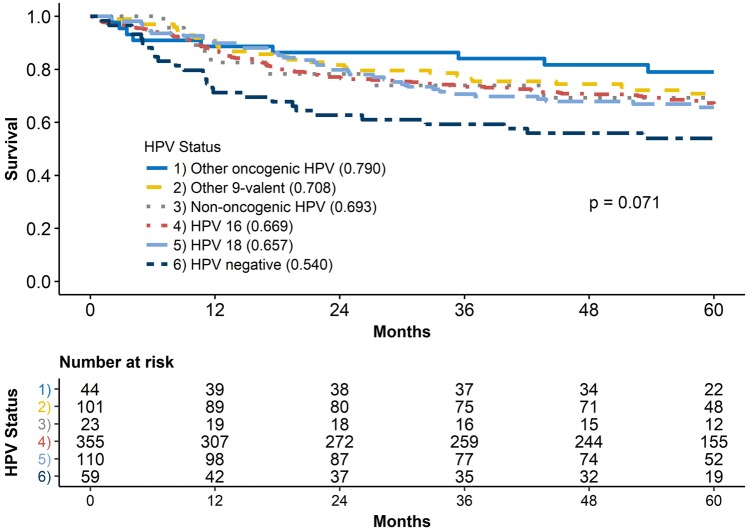
Unadjusted five-year all-cause survival by HPV hierarchy among invasive cervical cancer patients. *P* values were calculated using a two-sided log-rank test. HPV = human papillomavirus.

**Figure 2.**
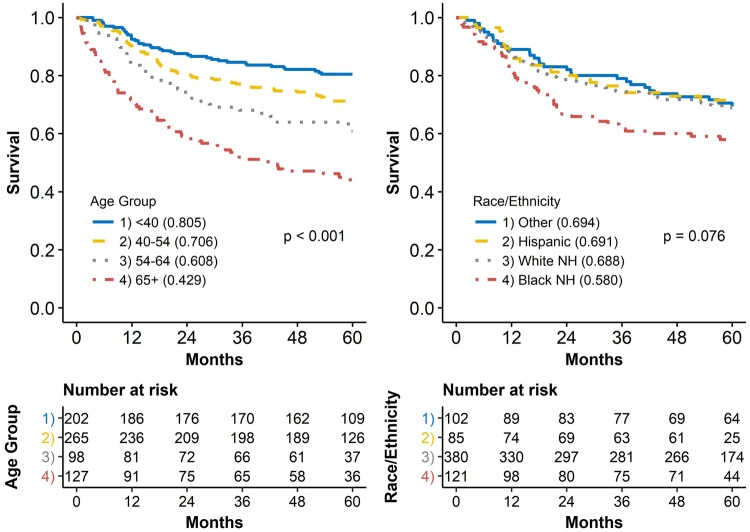
Unadjusted five-year all-cause survival among invasive cervical cancer patients by age (**left**) and race/ethnicity (**right**). *P* values were calculated using a two-sided log-rank test. NH = non-Hispanic.

**Figure 3.**
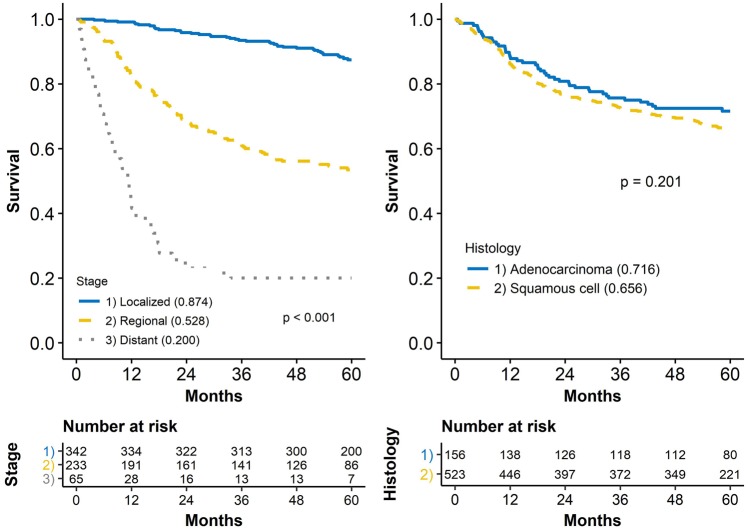
Unadjusted five-year all-cause survival among invasive cerivical cancer patients by stage (**left**) and histology (**right**). *P* values were calculated using a two-sided log-rank test.

Unadjusted five-year all-cause survival rates varied by HPV status: HPV 16: 66.9%, HPV 18: 65.7%, HPV 31/33/45/52/58: 70.8%, other oncogenic HPV genotypes: 79.0%, nononcogenic HPV genotypes: 69.3%, HPV-negative: 54.0% (*P* = .0710) (Figure 1). Five-year all-cause survival statistically significantly decreased with increasing age (Figure 2). Non-Hispanic black women had a statistically nonsignificantly lower five-year survival rate (58.0%) compared with other races (68.8%–69.4%) (Figure 2). More advanced tumor stages were statistically significantly associated with poorer survival (Figure 3), as were more advanced grades (data not shown). Tumor histology did not have a statistically significant impact on survival with squamous cell carcinomas (65.6%) and adenocarcinomas (71.6%) exhibiting similar five-year survival rates (Figure 3).

HPV genotype was associated with five-year all-cause survival following multivariable adjustment (Table 4). ICC patients positive for HPV 18 (hazard ratio [HR] = 1.00, 95% confidence interval [CI] = 0.64 to 1.55) and women with no detectable HPV (HR = 1.10, 95% CI = 0.63 to 1.94) had similar survival to women with HPV 16–positive tumors. Women with detectable HPV 31/33/33/45/52/58 had a statistically significant 40% reduced hazard of death at five years (HR = 0.60, 95% CI = 0.38 to 0.95), and women who tested positive for nononcogenic HPV genotypes had a statistically significant 57% reduced hazard of death at five years when compared with women with HPV 16 tumors (HR = 0.43, 95% CI = 0.19 to 0.96). Increasing age, advanced SEER summary stage, and more aggressive tumor grades were associated with statistically significantly increased five-year risk of death. Black non-Hispanic women had statistically significantly lower survival compared with women in the “other” race group (HR = 2.00, 95% CI = 1.22 to 3.29). There were no other statistically significant differences in adjusted survival between race/ethnicity groups. In unadjusted models, a statistically significant interaction was observed between HPV type and tumor histology (Supplementary Figure 1, available online). However, after adjustment, tumor histology and the interaction term (excluded from the final model) were no longer statistically significant predictors of survival. Surgery and chemotherapy were associated with increased five-year all-cause survival (HR = 0.48, 95% CI = 0.32 to 0.70, and HR = 0.49, 95% CI = 0.34 to 0.70, respectively).

**Table 4.** Multivariable Cox proportional hazards model* predicting five-year all-cause survival among invasive cervical cancer patients (n = 604)†

Characteristic	Wald χ^2^	DF	*P*	Adjusted hazard ratio (95% CI)
HPV hierarchy	11.01	5	.0513	
HPV 18 vs HPV 16				1.00 (0.64 to 1.55)
HPV 31/33/45/52/58 vs HPV 16				0.60 (0.38 to 0.95)
Other oncogenic HPV types vs HPV 16				0.52 (0.25 to 1.09)
Nononcogenic HPV types vs HPV 16				0.43 (.19 to 0.96)
HPV-negative vs HPV 16				1.10 (0.63 to 1.94)
Age at diagnosis	24.72	1	<.0001	
Per 5-y increase				1.14 (1.08 to 1.19)
SEER summary stage	82.77	2	<.0001	
Regional vs local				3.25 (2.04 to 5.17)
Distant vs local				11.41 (6.67 to 19.54)
Grade	6.20	2	.0450	
Moderately differentiated vs well differentiated				4.46 (1.37 to 14.51)
Poorly/undifferentiated vs well differentiated				4.18 (1.33 to 13.19)
Race/ethnicity	7.78	3	.0508	
Black non-Hispanic vs white non-Hispanic				1.40 (0.96 to 2.03)
Hispanic vs white non-Hispanic				0.96 (0.55 to 1.68)
Other vs white non-Hispanic				0.70 (0.45 to 1.09)
Histology type	0.61	1	0.4334	
Adenocarcinoma vs squamous cell				1.19 (0.77 to 1.82)
Surgery‡	13.90	1	.0002	0.48 (0.32 to 0.70)
Chemotherapy‡	15.55	1	.0001	0.49 (0.34 to 0.70)
Radiation‡	3.42	1	.0645	1.47 (0.98 to 2.22)

* Covariate effects and hazard ratios adjusted for all other variables in the table. CI = confidence interval; DF = Degrees of Freedom; HPV = human papillomavirus; SEER = Surveillance, Epidemiology, and End Results.

† Eighty-nine individuals were excluded from the multivariable analysis due to missing treatment status or treatment timing data (n = 604 with 192 events).

‡ Surgery, radiation, and chemotherapy were modeled as time-dependent covariates. Due to the time-dependent nature of the treatment variables, observations missing treatment status or timing were excluded from the multivariable analysis.

## Discussion

In this study, our results suggest that HPV genotype may influence survival in women with cervical cancer. After adjusting for patient demographics, tumor characteristics, and treatment, women with detectable HPV 31/33/45/52/58 and women who tested positive for nononcogenic HPV genotypes had a survival advantage when compared with women with HPV 16–positive tumors. No statistically significant differences in survival were observed for women with HPV 16–positive tumors when compared with women with tumors that were positive for HPV 18, other oncogenic types, or undetectable HPV. Women with HPV-negative tumors, which were primarily adenocarcinomas, had the poorest unadjusted five-year survival overall, but only represented 8.5% of the ICC cases in this study (5,25).

Aligning with the work of others, we observed no statistically significant difference in five-year all-cause survival in women with HPV 16–positive tumors compared with women with HPV 18–positive tumors after adjustment for other covariates (19,20). The improved survival observed among HPV 31/33/45/52/58 when compared with HPV 16 in our final multivariable model aligns with that reported by de Cremoux et al. (19), which showed improved survival among intermediate HPV risk types (31/33/35/39/52/58/59/73) when compared with HPV 16/18/45. Although not statistically significant in the final multivariable model, the poorest survival observed among women with HPV-negative ICCs aligns with other work that identified this trend in preliminary but not final models (20).

Our results are inconsistent with those from a representative cohort of Scottish women with cervical cancer that showed that the presence of HPV 16/18 in tumors predicted improved survival when compared with tumors that were not HPV 16/18–positive (26). In that study, unadjusted survival was poorer for women with HPV 16– or HPV 18–positive cervical cancer than for women with other oncogenic and nononcogenic HPV genotypes, although not statistically significantly so. The results may have been confounded because 40% of the comparison group had HPV-negative tumors. In the Scottish study, when women with HPV-negative tumors were omitted from the comparison group and presented separately in Kaplan-Meier plots, the HPV-negative group exhibited the lowest observed survival, rather than the HPV 16/18 ICC patients (26). Another study of 1067 ICC patients found that only HPV 18 was a statistically significant predictor of improved prognosis. However, this study was limited to women with stage I–IIA tumors who had undergone surgery as their primary treatment (27). An additional study suggested that HPV 16 was a statistically significant predictor of improved survival, but the analysis was limited to women who survived at least two months after completing their treatment regimen (28).

Women with ICCs that were positive for nononcogenic HPV types exhibited similar survival when compared with women with oncogenic HPV genotypes. Given the poor survival observed among women with HPV-negative tumors, this finding suggests that nononcogenic types may have some influence on patients’ survival following ICC diagnosis, although this observation was based on a limited sample (23 women with nononcogenic types and 59 with no HPV detected). Although HPV types in this group are currently classified as nononcogenic, a number of nononcogenic HPV types are from the high-risk alpha clade, and it is possible with future research that these types will be classified as carcinogenic (29).

The poorer survival observed among HPV-negative ICC tumors in this study closely aligns with the poorer survival observed among HPV-negative oropharyngeal tumors when compared with HPV-positive tumors (30,31). The frequency of HPV-negative ICC tumors (8.5%), however, is statistically significantly lower than the frequency of HPV-negative tumors in oropharyngeal cancers (28.5%) in the US population (30). It should be noted that the HPV-negative group could include 1) invasive cervical cancer cases that are truly HPV-negative, 2) samples in which HPV was not detected due to technical limitations, and 3) tumors of endometrial origin (5).

The results from this study demonstrate that HPV type influences the survival of ICC patients. However, the findings from this study are not strong enough to warrant implementation of HPV genotyping in the clinical setting. Future work will need to be conducted to determine how HPV genotyping may be used to modify treatment plans and inform prognosis. Additional research could include performing whole-genome next-generation sequencing to determine whether specific sublineages, variants, or single nucleotide polymorphisms are predictive of prognosis.

To our knowledge, this is one of the largest studies to analyze the impact that HPV genotype has on ICC patients’ survival. The large sample size and lengthy follow-up duration allowed us to assess the impact that HPV genotype has on survival while controlling for other important prognostic indicators in our models. One limitation is the calculation of survival time in this study based on all-cause mortality. Results could be different for disease-free survival or disease-specific mortality.

The results from this study support a growing body of evidence that HPV genotype may influence survival among ICC patients. After controlling for demographic, tumor, and treatment factors, women with HPV-negative tumors had the poorest survival overall but only represented 8.5% of the ICC in this study. Fortunately, among HPV-positive ICC cases, cancers associated with the poorest survival could have been prevented with the current bivalent, quadrivalent, or nonavalent HPV vaccinations. Further research is needed in well-annotated cohorts to define the role of HPV genotype on prognosis in women with ICC. Cervical cancer screening and HPV vaccination remain the most important interventions for the prevention of future cervical cancer cases.

## Funding

This work was supported in part by Centers for Disease Control and Prevention (CDC) grants 5U58DP000810-5 (Kentucky), 5U58DP000844-5 (Florida), 5U58DP000812-5 (Michigan), and 5U58DP000769-5 (Louisiana); and by the Surveillance, Epidemiology, and End Result (SEER) Program, National Institutes of Health, Department of Health and Human Services, under contracts N01-PC-35139 (Los Angeles), N01-PC-35143 (Iowa), and N01-PC-35137 (Hawaii). Collection of specimens from Kentucky, Florida, Michigan, and Louisiana, coordination of genotyping data from both the SEER registry and the National Program of Cancer Registries, and genotyping were largely supported by CDC intramural funds and Vaccine for Children Funds.

## Notes

Affiliations of authors: Centers for Disease Control and Prevention, Atlanta, GA (BDH, MS, TDT, ERU); University of Iowa, Iowa City, IA (CFL); University of Kentucky College of Public Health, Lexington, KY (TT); Michigan Department of Community Health, Lansing, MI (GC); University of Hawaii, Honolulu, HI (BYH); Health Science Center School of Public Health, Lousiania State University, New Orleans, LA (ESP); Department of Pathology, University of Florida College of Medicine, Gainesville, FL (EW); Cedars Sinai Medical Center, Los Angeles, CA (MTG).

The findings and conclusions in this report are those of the authors and do not necessarily represent the official position of the Centers for Disease Control and Prevention.

We would like to thank Dr. Stuart Massad for his review of the manuscript.

## Supplementary Material

Supplementary Data
