# Supplementary material for: Population-Based Assessment of HPV Genotype-Specific Cervical Cancer Survival: CDC Cancer Registry Sentinel Surveillance System
Source: JNCI Cancer Spectr. 2018 Aug 11;2(3):pky036. doi: 10.1093/jncics/pky036 (PMC6309887; doi:10.1093/jncics/pky036)

**Supplemental Figure 1**. Unadjusted Five-Year All-Cause Survival by HPV Hierarchy and Histology among Invasive Cervical Cancer Patients with HPV 16, HPV 18 or HPV-negative tumors.


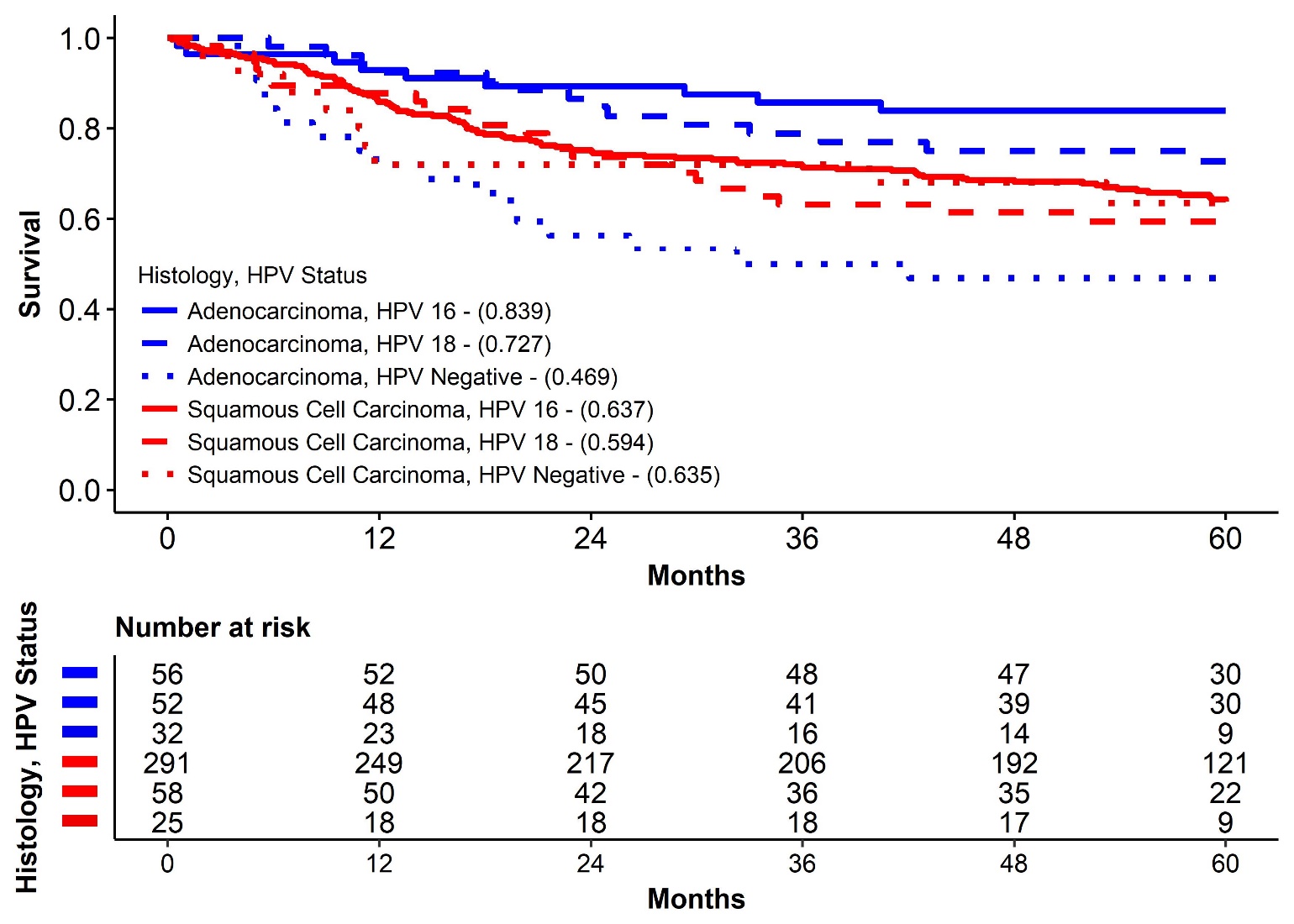

Supplement: Supplementary Data [file pky036_suppl.zip › Saraiya_Supplemental Figure File_jbFINAL.docx]
