# Supplementary figures and images for: Population-Based Assessment of HPV Genotype-Specific Cervical Cancer Survival: CDC Cancer Registry Sentinel Surveillance System
Source: JNCI Cancer Spectr. 2018 Aug 11;2(3):pky036. doi: 10.1093/jncics/pky036 (PMC6309887; doi:10.1093/jncics/pky036)

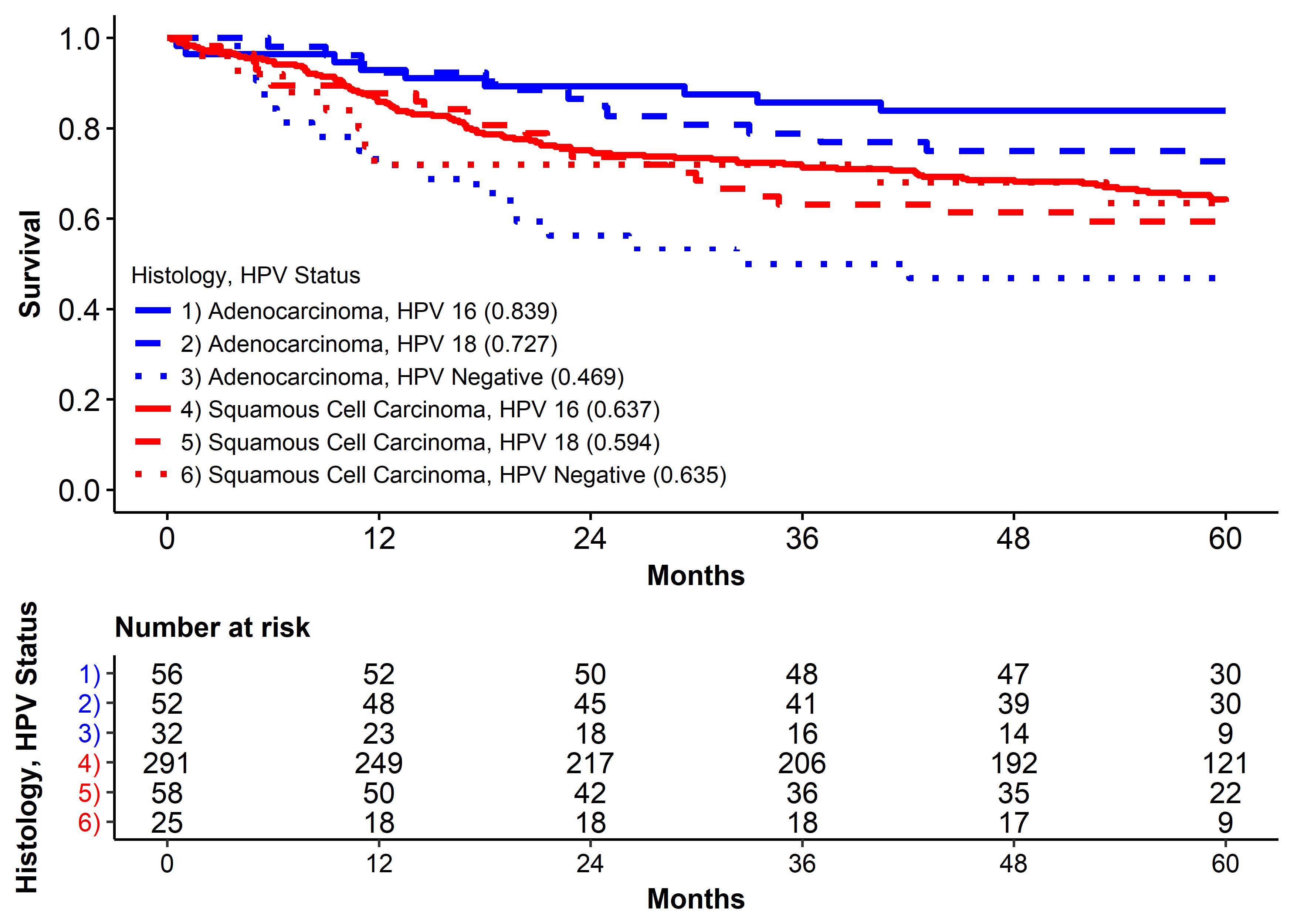

Supplement: Supplementary Data [file pky036_suppl.zip › SuppFigure_RiskLabel.jpg]
